# High-Throughput Genotyping of CRISPR/Cas Edited Cells in 96-Well Plates

**DOI:** 10.3390/mps1030029

**Published:** 2018-08-01

**Authors:** Lea Nussbaum, Jelena M. Telenius, Stephanie Hill, Priscila P. Hirschfeld, Maria C. Suciu, The WIGWAM Consortium, Damien J. Downes, Jim R. Hughes

**Affiliations:** 1 MRC Molecular Haematology Unit, Weatherall Institute of Molecular Medicine, Oxford OX3 9DU, UK; lea.nussbaum@lincoln.ox.ac.uk (L.N.); jelena.telenius@imm.ox.ac.uk (J.M.T.); stephanie.hill@ndcls.ox.ac.uk (S.H.); priscila.hirschfeld@ndcls.ox.ac.uk (P.P.H.); maria.suciu@genomicsplc.com (M.C.S.); 2 MRC WIMM Center for Computational Biology, Weatherall Institute of Molecular Medicine, Oxford OX3 9DU, UK; 3 Genomics plc, Oxford OX1 1JD, UK; 4 Weatherall Institute of Molecular Medicine, Oxford OX3 9DU, UK; jim.hughe@imm.ox.ac.uk; 5 Wellcome Trust Centre for Human Genetics, Oxford OX3 7BN, UK

**Keywords:** CRISPR/Cas9 genotyping, homology dependent repair, genome editing, genome-wide association study (GWAS) validation

## Abstract

The emergence in recent years of DNA editing technologies—Zinc finger nucleases (ZFNs), transcription activator-like effector (TALE) guided nucleases (TALENs), clustered regularly interspaced short palindromic repeats (CRISPR)/Cas family enzymes, and Base-Editors—have greatly increased our ability to generate hundreds of edited cells carrying an array of alleles, including single-nucleotide substitutions. However, the infrequency of homology-dependent repair (HDR) in generating these substitutions in general requires the screening of large numbers of edited cells to isolate the sequence change of interest. Here we present a high-throughput method for the amplification and barcoding of edited loci in a 96-well plate format. After barcoding, plates are indexed as pools which permits multiplexed sequencing of hundreds of clones simultaneously. This protocol works at high success rate with more than 94% of clones successfully genotyped following analysis.

## 1. Introduction

The past two decades have seen the explosion of available genomic editing tools for eukaryotic systems including Zinc finger nucleases (ZFNs) [1], transcription activator-like effector (TALE) guided nucleases (TALENs) [2], CRISPR/Cas nucleases [3,4,5], and most recently the clustered regularly interspaced short palindromic repeats (CRISPR)/Cas, and Base-Editors [6]. These systems operate via endogenous DNA repair mechanisms to bring about nucleotide changes and have greatly increased the routine throughput of genomic editing experiments. Generally, sequence changes arise during repair of double-strand DNA breaks, with repair primarily carried out by the non-homologous end joining (NHEJ) pathway, which generates small insertions or deletions (indels), and less commonly large deletions [7,8]. However, exact base-pair changes can also be generated by the less-frequent homology-dependent repair (HDR) pathway using co-transfected donor or template DNA [9]. While many experimental approaches have used pools of CRISPR/Cas guides to edit numerous loci, such as knock-out and drop-out screens [10,11,12,13], other experiments require a more tailored approach, using HDR to introducing specific changes within a few loci [9,14]. For example, the exact editing of single nucleotide polymorphisms (SNPs) within primary cells or cell lines is essential to functionally validate causative SNPs identified from genome-wide association studies [15].

Although methods for both editing cells and isolating clones of edited cells have become much more accessible, techniques for high-throughput screening of large numbers of clones are still required. These techniques are particularly important for projects where exact nucleotide changes are required as HDR occurs much less frequently than NHEJ, requiring larger numbers of clones to be screened to isolate correctly repaired loci [7,12,16]. While it is often possible to identify sequence changes via traditional Sanger sequencing these signals become confused by heterozygotes with small indels and are hard to incorporate into high-throughput pipelines; this is especially complex in polyploid cells with more than two alleles.

Conventional screening approaches use Sanger sequencing [17], which is low-throughput and poorly resolves heterozygotes. Alternatively, polymerase chain reaction (PCR)-capillary gel electrophoresis [18] and high-resolution melting-curve analysis [19] are high-throughput methods, but provide no sequence information. In contrast, next-generation sequencing generates signal from a single allele and makes signal deconvolution unnecessary for heterozygotes, while at the same time, allows high-throughput handling [20]. We have, therefore, developed a pipeline for the high-throughput genotyping of targeted loci in edited and single-cell sorted clones. Our method uses three rounds of amplification: first to isolate the locus of interest, second to barcode the well into which the clone was sorted, and third to index the specific plate. This method allows for several hundred clones to be genotyped simultaneously in a single sequencing run and also permits the multiplexed screening of editing at multiple loci. Importantly the protocol provides exact allelic sequence for resolution of complex alleles in diploid or polyploid cells and provides sequence files that can be analyzed with provided plateScreen96 scripts, or input into other analysis tools (e.g., OutKnocker [21]). This protocol will be highly useful for studies where isolation of clones with exact nucleotide changes is necessitated; and may be easily incorporated into an automated robotics system for even higher throughput applications.

## 2. Experimental Design

This protocol describes the genotyping of one or more loci of interest using next-generation sequencing. The protocol requires cells to have previously been edited, single-cell sorted and clonally expanded following a cell-type specific protocol (e.g., HEK-293 [22] or HUDEP-2 [23]). Expanded cells are split with one plate stored frozen and the other used for genotyping. As a control for this protocol it is appropriate to include one sample well from unedited cells. 

**Figure 1:**
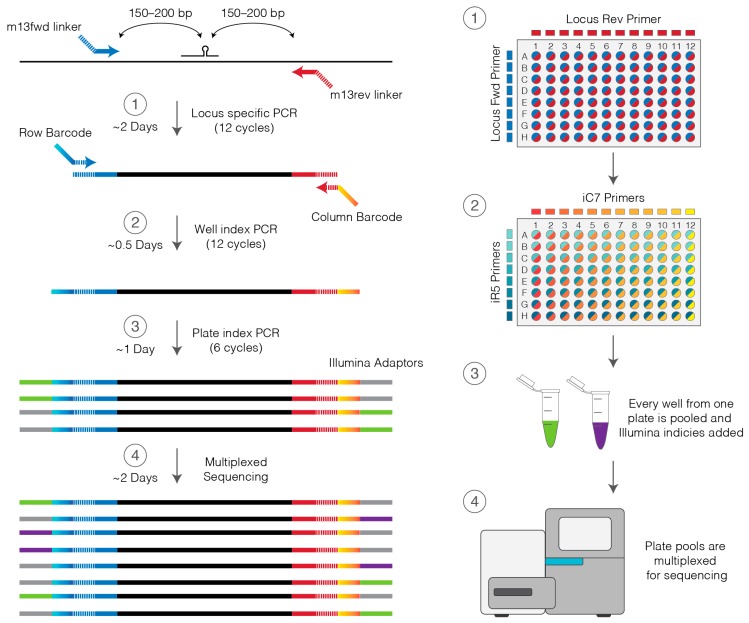
Workflow for 96-well plate amplification of edited loci from lysed cells (**1**), indexing of each well with a unique barcode primer pair (**2**), pooling of up to 96 wells from one plate for addition of Illumina adaptors and indices (**3**), followed by next-generation sequencing of pooled libraries from multiple plates (**4**). PCR: Polymerase chain reaction; fwd; forward; rev: reverse.

Genotyping (Figure 1) begins with the amplification of one or more loci of interest using specific primers which contain a linker sequence. The linker sequence is then used as a primer for a second round of amplification in which, maximally distinguishable combinations of barcodes are incorporated in a well-by-well basis. Each plate of PCR products is then pooled together for the addition of next-generation sequencing adaptors and indices, which allows multiplexing of numerous plates and the simultaneous sequencing of hundreds, or even thousands, of clones. Fastq files from sequencing are then analyzed using the plateScreen96 code [24,25] which reconstructs the original DNA fragments using overlapping forward and reverse reads (flashing). These flashed sequences are then mapped back to an appropriate genome build, and each unique allele along with how many times it was sequenced is reported. The output of plateScreen96 is an easily readable pdf report summarizing the genotypes of the genome edits in each well. Once the desired clones are identified these can be recovered from freezer storage and expanded.

### 2.1. Reagents and Materials

- Fetal bovine serum, FBS (Thermo Fisher, Paisley, UK; Cat. no.: 10270-106)
- Dimethyl sulphoxide, DMSO (VWR, Lutterworth, UK; Cat. no.: 23500.260)
- 96-well V-bottomed plates (Sigma-Aldrich, Dorset, UK; Cat. no.: CLS3894)
- Parafilm (VWR, Lutterworth, UK; Cat. no.: PM-996)
- 96-well PCR plate (Thermo Fisher; Cat. no.: AB1400L)
- Tris, 1 M, pH 8.0 (Thermo Fisher; Cat. no.: AM9855G)
- Ethylenediaminetetraacetic acid (EDTA), 0.5 mM, pH 8.0 (Thermo Fisher; Cat. no.: 15575-038)
- Tween 20, 50% (Thermo Fisher; Cat. no.: 003005)
- PCR grade water (Thermo Fisher; Cat. no.: AM9932)
- DNA low bind tubes (Eppendorf, Arlington, UK; Cat. no.: Z666548)
- Proteinase K (Thermo Fisher; Cat. no.: EO0491)
- Platinum PCR master mix (Thermo Fisher; Cat. no.: 12532016)
- Locus specific primers, 10 µM mix, see Section 2.3 (Sigma, St Louis, MO, USA or Integrated DNA Technologies (IDT), Skokie, Illinois, USA)
- Agarose (Roche, Burgess Hill, UK; Cat. no.: 11388983001)
- 10× Tris acetate-EDTA, TAE (Sigma-Aldrich; Cat. no.: 11666690001)
- 100 bp ladder (New England Biolabs, Hitchin, UK; Cat. no.: N0551G)
- Exonuclease I, *Escherichia coli* (New England Biolabs; Cat. no: M0293L)
- Shrimp alkaline phospatase, rSAP (New England Biolabs, Hitchin, UK; Cat. no.: M0371L)
- Custom iR5 and iC7 barcoded primers, Table 1 (Sigma or IDT)
- Agencourt Ampure XP SPRI Beads (Beckman Coulter, High Wycombe, UK; Cat. no.: A63881)
- 100% Ethanol (VWR, Lutterworth, UK; Cat. no.: 20821.330)
- Qubit BR DNA assay kit (Thermo Fisher; Cat. no.: Q32850)
- NEB Ultra II (New England Biolabs; Cat. no.: 7645S/L)
- PCR Tube (Appleton Woods, Birmingham, UK; Cat. no.: TA571)
- NEB Next Multiplex Oligos for Illumina primer set 1 (New England Biolabs; Cat. no.: E7500S/L)
- NEB Next Multiplex Oligos for Illumina primer set 2 (New England Biolabs; Cat. no.: E7335S/L)
- Herculase II Fusion polymerase kit (Agilent, Cheadle, UK; Cat. no.: 600677)
- Tris-EDTA, TE (Sigma-Aldrich; Cat. no.: 99302)
- D1000 reagents (Agilent; Cat. no.: 50675583)
- D1000 loading tips (Agilent; Cat. no.: 50675153)
- D1000 screen tape (Agilent; Cat. no.: 50675582)
- KAPA Library Quantification Complete Kit (Roche; Cat. no.: KK4824)
- MiSeq Reagent Nano Kit, v2 500-Cycles (Illumina, Cambridge, UK; Cat. no.: MS-103-1003)
- PhiX Control v3 (Illumina; Cat. no.: FC-110-3001)

### 2.2. Equipment

- 8- or 12-channel pipette (Labgene Scientific, Châtel-Saint-Denis, Switzerland: Cat. no.: 5121, 5125)
- Centrifuge with buckets for plates (Eppendorf; Cat. no.: 5810R)
- Thermocycler with 96-well plate capacity (Bio-Rad, Watford, UK; Cat. no.: T100)
- Electrophoresis gel tank and power pack
- Magnetic rack (DynaMag-2; Thermo Fisher; Cat. no.: 13221)
- Minifuge (Starlab, Milton Keynes, UK; Cat. no.: N2631-0007)
- Qubit fluorometer (Thermo Fisher; Cat. no.: Q33226)
- Agilent 2200 TapeStation (Agilent; Cat. no.: G2964AA)
- Real-time quantitative polymerase chain reaction (qPCR) thermocycler (Thermo Fisher StepOnePlus; Thermo Fisher; Cat. no.: 4376598)
- MiSeq (Illumina; Cat. no.: SY-410-1003)
- Microcentrifuge (Eppendorf; Cat. no.: 5424R)

### 2.3. Custom Locus Primers

The first PCR step of this protocol requires amplification of the edited locus using custom primers (Supplementary Materials Table S1). Primers are designed using standard tools such as IDT PrimerQuest [26] and should be positioned between 150–200 bp from the editing site to generate a 300–400 bp PCR product. When screening for HDR events is essential primers do not overlap any donor sequence (such as single stranded oligodeoxynucleotides, ssODNs) to avoid amplification from incorrect insertions. Additionally, distant primers minimize the likelihood of false-positive homozygotes generated by a large deletion on one allele removing a primer binding site. False homozygotes can be filtered either with a heterozygous SNP within the PCR product, or by screening for heterozygosity with a larger PCR product (>5 kb in size) to identify large deletions. However, placement of primers should be such that the final PCR product is no greater than ~400 bp, as this enables sequence coverage across the entire amplicon and generates overlapping reads to reconstruct the original PCR product, which is essential for complete analysis. Up to five primer pairs are designed and tested in silico with BLAT [27] to ensure site locus specificity. A modified m13fwd (5′-GTAAACGACGGCCAGT-3′) and m13rev (5′-AGCGGATAACAATTTCACACAGGA-3′) sequence are then added to the 5′ ends of the forward and reverse primers respectively. The m13 sequences serve dual purposes, acting as adaptors for barcode primer binding and allowing for traditional Sanger sequencing if necessary.

**Figure 2:**
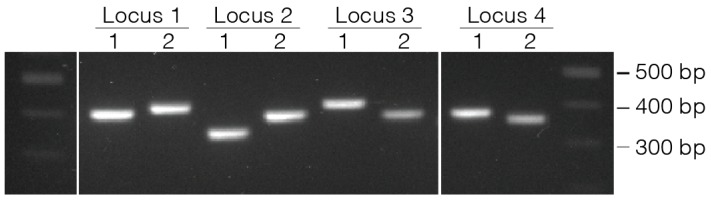
Two pairs of primers are validated for each edited locus with genomic DNA from the cells to be edited and running on a 2% agarose gel.

As the addition of the m13 linkers may alter the binding specificity of the primer, at least two primer pairs are ordered and tested on genomic DNA (Figure 2). The PCR products from these primers can be sequenced to confirm specificity if necessary. Primer-pair cocktails are made by mixing equal volumes of 20 µM forward and reverse primer dilutions to generate a working stock containing each primer at 10 µM.

### 2.4. Primers to Barcode Individual Wells

Locus specific PCR products are barcoded with one of eight forward primers (iR5) and one of twelve reverse primers (iC7) in all 96 permutations to uniquely identify each well (Figure 2). These primers contain a 3 bp spacer (GAT), and 8 bp barcode, and a modified m13fwd or m13rev sequence (Table 1) to prime from the locus specific PCR product. Primers should be made up to a working stock of 10 µM.

### 2.5. Analysis Software

The custom plateScreen96 scripts are available on GitHub [24], with additional test files, manual and system requirements also available online [25].

## 3. Procedure

Here we provide instructions for splitting non-adherent cells into two plates, one for freezing and storage (stock plate) and the other for lysis (genotype plate), amplification and indexing producing a next-generation sequence ready library (Figure 1). Cells should already have been edited, single-cell fluorescence-activated cell sorting (FACS) separated and expanded in a method appropriate for the cell type (e.g., HEK-293 [22]; HUDEP-2 [23]). Where single-cell FACS is not available, limiting dilution may be used but care should be made to ensure each well is occupied by a unique clone.

### 3.1. Clonal Expansion. Time for Completion: 2–3 Weeks

1. Using the appropriate growth media, clonally expand single-cell sorted colonies, splitting them as necessary until they occupy two to four wells of a 96-well plate at a high level of confluence (80–90%).

### 3.2. Splitting and Freezing Cells. Time for Completion: 3 h

2. Reduce media volume to <100 µL per well and mix by pipetting.
3. Prepare two new 96-well V-bottomed plates (one for stock storage and one for genotyping) by combining two wells of highly confluent cells. Note: If cells have only grown to occupy two wells of a flat-bottomed 96-well plate, transfer a single well to each V-bottomed plate.

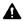
**CRITICAL STEP** Clones should occupy identical wells in both the stock and genotyping plates.

4. Set aside the genotyping plate and pellet the cells in the stock plate by centrifugation (250× *g*, 5 min, room temperature).
5. During this spin step, prepare 5 mL of sterile freezing media (90% FBS, 10% DMSO *v*/*v*) per stock plate.
6. After centrifugation use an 8- or 12-channel pipette to carefully remove supernatant from the pelleted cells in the stock plate.

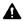
**CRITICAL STEP** Take care not to dislodge and discard cells by disturbing the pellet.

7. Quickly and carefully resuspend cells in 50 µL of freezing buffer.
8. Wrap the stock plate in parafilm, place in polystyrene box or freezing box and store at −80 °C.

### 3.3. Cell Lysis. Time for Completion: Overnight (18 h)

9. Prepare 3 mL lysis buffer per full plate by adding proteinase K (see Section 5).
10. Pellet cells in the genotyping plate from step 4 in Section 3.2 (250× *g*, 5 min, room temperature).
11. Using an 8- or 12-channel pipette remove supernatant and resuspend in 30 µL lysis buffer.
12. Seal plate and incubate at 37 °C overnight.
13. On the following day, heat to 95 °C for 10 min in thermocycler to deactivate proteinase K.
14. Cool to 4 °C

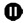
**PAUSE STEP** Lysed cells can be stored at −20 °C for up to one month or at −80 °C for one year.

### 3.4. Locus Amplification and PCR Product Clean-Up. Time for Completion: 4 h

15. On ice, prepare 1150 µL of Locus PCR master mix per lysed plate by combining 1125 µL Platinum master mix and 25 µL of 10 µM locus primer mix (Section 2.3).
16. Aliquot 11.5 µL of Locus PCR master mix into each well of a 96-well PCR plate on ice.
17. On ice, add 1 µL of cell lysate to the corresponding well of the PCR plate and mix by pipetting.
18. Place in thermocycler and amplify using the Platinum PCR cycling settings in Table 2.
19. Transfer 1 µL of PCR products to the corresponding well of a new 96-well PCR plate.
20. Prepare 220 µL PCR clean-up master mix per plate by combining 16.5 µL exonuclease I, 16.5 µL shrimp alkaline phosphatase (SAP) and 187 µL PCR grade water.
21. Add 2 µL of PCR clean-up master mix to each well, mix by pipetting.
22. Incubate in a thermocycler at 37 °C for 30 min, 85 °C for 15 min, and then cool to 4 °C.

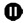
**PAUSE STEP** After stopping the reaction, the mix can be stored at 4 °C overnight.

### 3.5. Well Barcoding and PCR Product Clean-Up. Time for Completion: 3 h

23. To each well of cleaned-up PCR product add 11.5 µL Platinum master mix (perform on ice).
24. Prepare a stock 96-well plate of barcoding primers by combining all unique pairs of row and column primers at 5 µM each by adding equal volumes (2–5 µL) of each iC7 and iR5 primer at 10 µM. This primer plate may be stored at −20 °C and used multiple times.
25. Using the barcoding primers prepared in step 24 and a multichannel pipette add 0.5 µL of primers to the appropriate wells of the genotyping plate.
26. Place in a thermocycler and amplify using the Platinum PCR cycling settings in Table 2.
27. During amplification bring an aliquot of 875 µL Ampure XP beads to room temp in an Eppendorf tube.
28. Pool 5 µL from each well of a single plate into a single 1.5 mL DNA low bind tube (480 µL in total). Store excess PCR reaction at −20 °C.
29. Add 864 µL Ampure XP beads (1.8 volumes) to the pooled PCR products. Mix by pipetting.
30. Incubate at room temp for 5 min.
31. During incubation prepare 80% ethanol (1200 µL 100% ethanol, 300 µL PCR grade water).
32. Place on magnetic stand. After the liquid has cleared ~5 min), remove and discard the liquid without disturbing the beads.
33. Add 700 µL of fresh 80% ethanol again without disturbing beads. Incubate 30 s.
34. Remove the ethanol and the repeat wash with another 700 µL of fresh 80% ethanol.
35. Discard the ethanol, spin briefly on a bench-top centrifuge and replace on magnetic stand.
36. Discard the residual ethanol and allow to air dry until beads appear matte in appearance ~5 min).

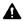
**CRITICAL STEP** Do not over dry the beads as this will reduce yield. Beads should appear like damp mud, neither glossy wet nor dry. Cracks in the bead pellet are indicative of over-drying.

37. Remove tube from magnet and resuspend beads in 55 µL of PCR grade water by pipetting 10 times.
38. Incubate at room temp for 2 min.
39. Replace on magnetic stand.
40. Once clear (~4 min) recover 53 µL of eluted PCR product and transfer to a new 1.5 mL DNA low-bind tube.
41. Use 2 µL of eluted PCR to quantify the DNA concentration using a Qubit BR DNA kit.
42. Use 1 µL of eluted PCR to evaluate the product size on a D1000 TapeStation gel.

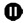
**PAUSE STEP** After purification the products can be stored at 4 °C overnight or at −20 °C for several months.

### 3.6. Addition of Illumina Adaptors. Time for Completion: 3 h

These steps follow the NEB Ultra II protocol with minor modifications.

43. Combine 50 µL of DNA (0.5–2 µg), 7 µL 10× End Prep Buffer and 3 µL End Prep Enzyme. Mix by pipetting and incubate at 20 °C for 30 min in a thermocycler (lid open or un-heated).
44. Increase temperature to 65 °C and incubate for 30 min (lid heated to 75 °C).
45. Add 30 µL Ultra II Ligation Master Mix, 2.5 µL NEBNext Adaptor, 1 µL Ligation enhancer. Mix by pipetting and incubate at 20 °C for 15 min.
46. Add 3 µL USER^TM^ enzyme. Mix by pipetting and incubate at 37 °C for 15 min.
47. During this USER^TM^ enzyme reaction warm 90 µL of Ampure XP beads to room temp.
48. Add 87 µL Ampure XP beads (0.9×) and mix by pipetting. Incubate at room temp for 5 min.
49. During incubation prepare 80% ethanol (800 µL 100% ethanol, 200 µL PCR grade water).
50. Place on magnetic stand. Without disturbing beads, discard liquid when clear (~5 min).
51. Add 500 µL of fresh 80% ethanol without disturbing beads. Incubate 30 s.
52. Remove ethanol and repeat wash with another 500 µL of fresh 80% ethanol.
53. Discard ethanol, spin briefly on a bench-top centrifuge and replace on magnetic stand.
54. Discard residual ethanol and allow to air dry until beads are matte in appearance (~5 min).

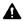
**CRITICAL STEP** Do not over dry the beads as this will reduce yield. Beads should appear like damp mud, neither glossy wet nor dry. Cracks in the bead pellet are indicative of over-drying.

55. Remove tube from magnet and resuspend beads in 30.5 µL of PCR grade water by pipetting 10 times.
56. Incubate at room temperature for 2 min.
57. Replace on magnetic stand.
58. Once clear ~4 min) recover 28.5 µL of eluted PCR product and transfer to a new PCR tube.

### 3.7. Indexing for Multiplexing of Multiple Plates. Time for Completion: 2 h

59. To the adaptor ligated library add:
  - 5 µL NEB Universal Primer
  - 5 µL NEB Index Primer
  - 10 µL Herculase II 5× Buffer
  - 0.5 µL deoxynucleotide triphosphate (dNTP)
  - 1 µL Herculase II Polymerase
60. Mix by pipetting and amplify using the Herculase PCR cycling settings in Table 3.
61. During the PCR warm 40 µL of Ampure XP beads to room temp.
62. Add 40 µL Ampure XP beads (0.8×) to the PCR and mix by pipetting. Incubate at room temperature for 5 min.
63. During incubation prepare 80% ethanol (800 µL 100% ethanol, 200 µL PCR grade water).
64. Place on magnetic stand. Without disturbing beads, discard liquid when clear ~5 min).
65. Add 500 µL of fresh 80% ethanol without disturbing beads. Incubate 30 s.
66. Remove ethanol and repeat wash with another 500 µL of fresh 80% ethanol.
67. Discard ethanol, spin briefly on a bench-top centrifuge and replace on magnetic stand.
68. Discard residual ethanol and allow to air dry until beads are matte in appearance (~5 min).

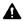
**CRITICAL STEP** Do not over dry the beads as this will reduce yield. Beads should appear like damp mud, neither glossy wet nor dry. Cracks in the bead pellet are indicative of over-drying.

69. Remove tube from magnet and resuspend beads in 30 µL of 0.1× Tris-EDTA (TE) by pipetting 10 times.
70. Incubate at room temperature for 2 min.
71. Replace on magnetic stand. Once clear (~4 min) recover 28 µL of eluted PCR product and transfer to a new DNA low bind tube.
72. Confirm PCR product size using either a 2% agarose gel or D1000 TapeStation.

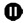
**PAUSE STEP** After this step the indexed library can be stored at 4 °C overnight or at −20 °C for several months.

### 3.8. Quantification and Sequencing. Time for Completion: 28 h

73. Make a 1:10,000 and 1:20,000 dilution of indexed PCR products and quantify by real-time qPCR using the KAPA Illumina quantification kit.
74. If sequencing in-house prepare 8 nM dilutions of each PCR plate pool and combine differently indexed plates in equal volumes.
75. If sequencing in a core facility prepare DNA pools to their specifications.
76. Sequence on a MiSeq using a 500-cycle Nano kit (250 bp reads, paired end) with 10% PhiX.

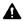
**CRITICAL STEP** Locus-specific PCR products will have near identical sequences, PhiX is essential to avoid MiSeq run failure from low complexity. If sequencing a single locus specific PCR product, increase PhiX to 30%.

### 3.9. Data Analysis. Time for Completion: 1–3 Days

All scripts as well as sample files and fastq files (analyzed below) are available via GitHub as a user tutorial.

77. Download fastq files from either BaseSpace or core facility provider.
78. In a single UNIX directory prepare the following metadata plain text files:
  1. *PIPE_fastqPasths.txt*: Containing names and paths to fastq files.
  2. *PIPE_spacerBarcodePrimer_FWD.txt*: Containing forward primer sequences.
  3. *PIPE_spacerBarcodePrimer_REV.txt*: Containing reverse primer sequences.
  4. *PIPE_targetLocus_xxX.bed*: Locus PCR amplification coordinates and additional region(s) of interest highlighted (Note: species of interest replaces xxX in the file name, e.g., PIPE_targetLocus_hg19.bed)

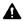
**CRITICAL STEP** Plain text files are essential as rich text files will not be read correctly and cause analysis failure.

79. Run analysis with the following minimal command (additional parameters are available): > ./plateScreen96.sh-g hg19.
80. Analyze pipe output and select clones to recover (see Expected Results).

### 3.10. Recovery of Clones of Interest. Time for Completion: 2 h

81. Prepare one 1.5 mL microcentrifuge tube per clone by adding 1 mL sterile phosphate-buffered saline (PBS).
82. Recover plate from −80 °C storage and remove parafilm.
83. Working quickly, add 150 µL of sterile PBS to wells containing clones to be recovered.
84. As each clone thaws, pipette mix to quickly combine with PBS, transfer melted clones to microcentrifuge tubes containing PBS.
85. Centrifuge to pellet cells (250× *g*, 5 min).
86. Discard supernatant and resuspend in appropriate amount of growth media and plate for expansion.

## 4. Expected Results

**Figure 3:**
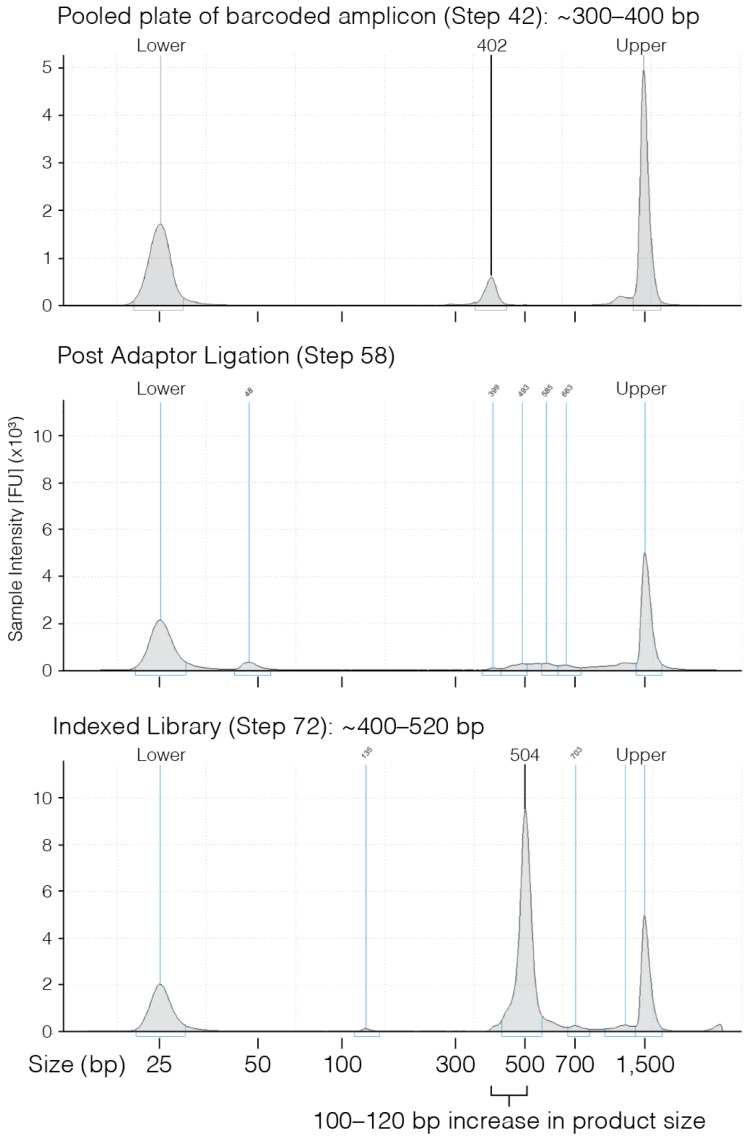
D1000 TapeStation traces of the barcoded amplicon, adaptor ligated fragments, and indexed amplicon show the increasing size of the DNA fragment with indexing. FU: Fluorescent units.

**Figure 4:**
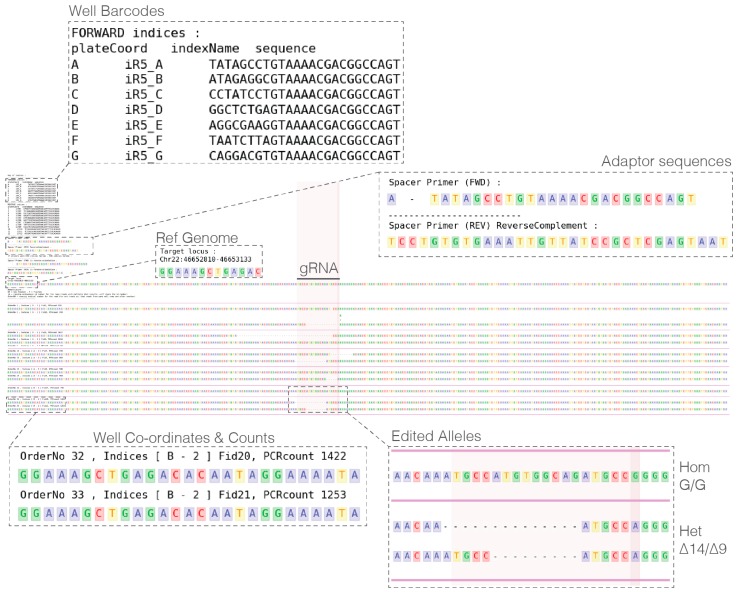
PlateScreen96 generates a PDF of a colored alignment of reads against the reference genome and reports input barcode and adaptor sequences and allows highlighting of key regions such as the guide RNA (gRNA). Reads are sorted per identified well and counts given, allowing users to assign genotypes for edited alleles. In this example, rs4508712 was edited from homozygous A/A to homozygous G/G.

During amplification and indexing of the edited locus, quality-control checks that are performed ensure libraries are successfully prepared for sequencing (Figure 3). Analysis of sequenced clones using plateScreen96 custom scripts will provide numerous metadata files including sequence quality, metrics of flashing, and importantly a PDF/PNG report (Figure 4). The report includes important metadata from the analysis, including barcode and adaptor sequences, reference genome coordinates and sequence, optional cell line sequence, and finally reads from each clone sorted by well and reporting PCR number. The aligned reads allow manual inspection to determining editing outcome and identify clones of interest. Although performing at a high success rate, not all wells generate sufficient sequence (threshold 10 reads), to be reported (Table 4). The success of sequencing is highly dependent on the number of cells used at the initial lysis step, with two near confluent wells from a 96-well plate providing the optimal results.

## 5. Reagents Setup

### Cell Lysis Buffer

Lysis Buffer (Table 5) can be made in advance by excluding proteinase K and stored at 4 °C for up to one month. Proteinase K should be added fresh on the day of use.

## Figures and Tables

**Table 1:** Oligonucleotides for barcoding 96-wells.

ID	Barcode	Sequence
		**iR5 – Row Barcode Primers (Forward)**
iR5_A	TATAGCCT	GATTATAGCCTGTAAAACGACGGCCAGT
iR5_B	ATAGAGGC	GATATAGAGGCGTAAAACGACGGCCAGT
iR5_C	CCTATCCT	GATCCTATCCTGTAAAACGACGGCCAGT
iR5_D	GGCTCTGA	GATGGCTCTGAGTAAAACGACGGCCAGT
iR5_E	AGGCGAAG	GATAGGCGAAGGTAAAACGACGGCCAGT
iR5_F	TAATCTTA	GATTAATCTTAGTAAAACGACGGCCAGT
iR5_G	CAGGACGT	GATCAGGACGTGTAAAACGACGGCCAGT
iR5_H	GTACTGAC	GATGTACTGACGTAAAACGACGGCCAGT
		**iC7 – Column Barcode Primers (Reverse)**
iC701	ATTACTCG	GATATTACTCGAGCGGATAACAATTTCACACAGGA
iC702	TCCGGAGA	GATTCCGGAGAAGCGGATAACAATTTCACACAGGA
iC703	CGCTCATT	GATCGCTCATTAGCGGATAACAATTTCACACAGGA
iC704	GAGATTCC	GATGAGATTCCAGCGGATAACAATTTCACACAGGA
iC705	ATTCAGAA	GATATTCAGAAAGCGGATAACAATTTCACACAGGA
iC706	GAATTCGT	GATGAATTCGTAGCGGATAACAATTTCACACAGGA
iC707	CTGAAGCT	GATCTGAAGCTAGCGGATAACAATTTCACACAGGA
iC708	TAATGCGC	GATTAATGCGCAGCGGATAACAATTTCACACAGGA
iC709	CGGCTATG	GATCGGCTATGAGCGGATAACAATTTCACACAGGA
iC710	TCCGCGAA	GATTCCGCGAAAGCGGATAACAATTTCACACAGGA
iC711	TCTCGCGC	GATTCTCGCGCAGCGGATAACAATTTCACACAGGA
iC712	AGCGATAG	GATAGCGATAGAGCGGATAACAATTTCACACAGGA

**Table 2:** Platinum polymerase chain reaction (PCR) Amplification.

Step	Temp.	Time
Step 1	94 °C	2 min
Step 2	94 °C	30 s
Step 3	62 °C	30 s
Step 4	68 °C	1 min
Repeat steps 2–4 for a total of 12 cycles		
Step 5	72 °C	10 min
Step 6	4 °C	∞

**Table 3:** Herculase PCR Amplification.

Step	Temp.	Time
Step 1	98 °C	30 s
Step 2	98 °C	10 s
Step 3	65 °C	30 s
Step 4	72 °C	30 s
Repeat steps 2–4 for a total of 6 cycles		
Step 5	72 °C	5 min
Step 6	4 °C	∞

**Table 4:** Example results for screening of edited erythroid cells.

	Wells Processed	Successfully Sequenced	Success Rate
Locus 1	52	49	94.2%
Locus 2	244	217	88.9%
Locus 3	87	85	97.7%
Locus 4	59	53	89.8%
Locus 5	47	43	91.4%
Locus 6	74	72	97.2%
Locus 7	236	234	99.1%
Total	799	753	94.2%

**Table 5:** Sufficient lysis buffer for one full 96-well plate.

Reagent	Volume	Final Conc.
1 M Tris, pH 8.0	150 µL	50 mM
0.5 M EDTA, pH 8.0	6 µL	1 mM
50% Tween 20	30 µL	0.5%
PCR Grade Water	2.805 mL	-
Proteinase K (>0.6 U/µL)	9 µL	>0.0018 U/µL

## Supplementary Materials

The following are available online at http://www.mdpi.com/2409-9279/1/3/29/s1, Table S1: List of primers used to screen for editing.
